# Esophageal Dysbiosis in Achalasia and Cancer Development: A Critical Review

**DOI:** 10.3390/genes14081521

**Published:** 2023-07-26

**Authors:** Francisco Tustumi, Vitor Pelogi Arienzo, Isabela Roskamp Sunye, Phellipe Fabbrini Santos Lucas, Bárbara Buccelli Colonno, Julia Grams Quintas, Elis Nogara Lisboa, Daniel José Szor

**Affiliations:** Department of Health Sciences, Hospital Israelita Albert Einstein, Sao Paulo 05652-900, Braziljuliagquintas@gmail.com (J.G.Q.);

**Keywords:** achalasia, esophagus, squamous cell carcinoma, carcinoma, neoplasm, dysbiosis

## Abstract

Background: Microorganisms provide various benefits to their human hosts, including assisting with digestion, synthesizing certain vitamins, developing the gastrointestinal and immune systems, regulating metabolism, and protecting against some pathogens. However, microbial imbalances can cause tissue damage and contribute to inflammatory disorders and cancers. Microbial dysbiosis refers to an imbalance or disruption in the normal composition and function of the microbial communities that inhabit various body parts, including the gut, oral cavity, skin, and reproductive tract. Emerging research suggests that microbial dysbiosis plays a significant role in cancer development and progression. This issue is particularly relevant in achalasia, in which food stasis, changes in endoluminal pH, and poor esophageal clearance might contribute to esophageal microbial dysbiosis. This study aimed to evaluate the association between dysbiosis and esophageal cancer development, focused on esophageal dysmotility disorders. Methods: This study is a critical review, gathering the current evidence for the association between dysbiosis and the development of esophageal cancer. Results: Studies have shown that microbiota play a role in cancer development, although the mechanisms for how they do so are not yet fully understood. One possible explanation is that microbiota alterations can lead to chronic inflammation, promoting cancer cell growth. Additionally, some bacteria produce toxins that can damage DNA and cause genomic instability, and certain bacterial products can promote tumor growth. Conclusion: Despite the close relationship between dysbiosis and cancer development in esophageal dysmotility disorders, further investigations are still needed to elucidate the precise mechanisms by which dysbiosis contributes to cancer development and to identify potential therapeutic interventions targeting the microbiota to prevent or treat cancer.

## 1. Introduction

Since its first description in 1672, achalasia has been one of the most studied motor diseases of the esophagus. Derived from the Greek word a-khalasis, its main physiopathological event is literally the “lack of relaxation” of the lower esophageal sphincter during deglutition, together with the impaired peristalsis of the esophageal body [1]. Although not yet completely understood, the mechanisms leading up to both events may be the neuronal degeneration of the myenteric plexus or the reduced density of Cajal cells in the gastroesophageal junction. Also, some new evidence points towards the role of the inflammatory process and disruption of inhibitory neurons, releasing nitric oxide. Exposure to environmental influences, infection, and genetic abnormality are among theories that still lack global literary acceptance [2,3].

Achalasia is a rare disease affecting less than 1% of the population, presenting similarly in both genders and among races, mostly between 30 to 60 years old. Although most cases are idiopathic, in South America, especially in Brazil, the diagnosis has augmented epidemiological relevance as it is frequently associated with Chagas disease [1,2].

The diagnosis is commonly delayed due to its confusion with gastroesophageal reflux disease (GERD). The classical presentation includes progressive dysphagia for solids and liquids, regurgitation of undigested food or saliva, vomiting, and weight loss [3].

There are three subtypes of achalasia distinguished by manometric assessment. Failed lower esophageal sphincter (LES) contractions and no esophageal pressurization characterize achalasia type 1. Type 2 is a pan-esophageal pressurization occurring with at least 20% of swallows. Type 3 is the presence of premature contraction for at least 20% of the swallows, with premature contraction defined as distal latency <4.5 s [2].

The types 1 and 2 are explained by the loss of ganglion cells, which is more severe in type 1. In type 3, inhibitory neuron function is damaged, making it less effective. Cytokine-induced alterations in gene expression possibly mediate this situation [2].

Achalasia is a known risk factor for esophageal cancer, both squamous cell carcinoma and adenocarcinoma. The first record to point out a possible association between achalasia and cancer is dated 1872, in a case report of a benign esophageal condition associated with cancer [4]. More recently, Sato et al. analyzed a large cohort of 2714 achalasia patients and demonstrated that the risk of developing cancer in patients with achalasia was 16.7 times higher for men and 8.8 times higher for women [5].

The mechanisms involving esophageal dysmotility and cancer have been widely studied. Food stasis and its continuous digestion by saliva enzymes probably have a massive role in inducing chronic inflammation [6,7]. However, the exact mechanisms are not entirely understood, and many factors might synergistically contribute to cancer development. In this setting, the interaction between the esophagus epithelium and its microbiome can be a cornerstone contributing factor [8]. Food stasis, changes in endoluminal pH, and poor esophageal clearance might contribute to esophageal microbial dysbiosis in achalasia [9]. The overgrowth of certain bacteria strains might promote carcinogenic effects by causing mutagenesis, angiogenesis, and inhibition of apoptosis [9].

Microbial dysbiosis refers to an imbalance or disruption in the normal composition and function of the microbial communities that inhabit various parts of the body, including the gut, oral cavity, skin, and reproductive tract. Emerging research suggests that microbial dysbiosis plays a significant role in cancer development and progression.

The present study’s objective is to critically review the association between esophageal dysbiosis and cancer development.

## 2. Material and Methods

This is a critical review that gathers scientific evidence for esophageal dysbiosis and cancer development. We provide a critical analysis of the current literature for researchers and clinicians to understand physiological mechanisms for cancer development, providing tools for future studies in the area.

The following search terms were used: “dysbacterioses”, “disbacterioses”, “dysbiosis”, “disbiosis”, “esophageal”, “esophagus”, “neoplasms”, “cancer”, “malignant”. The search was performed in the electronic databases PubMed, Lilacs/BVS, Embase, Scopus, Web of Science, and Google Scholar. This review included observational or experimental human study designs or in vivo or in vitro models.

## 3. Results

### 3.1. Carcinogenesis

Dysbiosis has been linked to the development of several types of cancer, including colorectal, gastric, liver, pancreatic, and breast cancer. Specific bacterial strains or dysbiotic patterns have been identified in association with these cancers, highlighting the potential role of the microbiota in their pathogenesis. Consequently, dysbiosis related to esophageal stasis probably has a significant role in the carcinogenesis of esophageal cancer.

#### 3.1.1. Microbiota and Carcinogenesis

**Figure 1:**
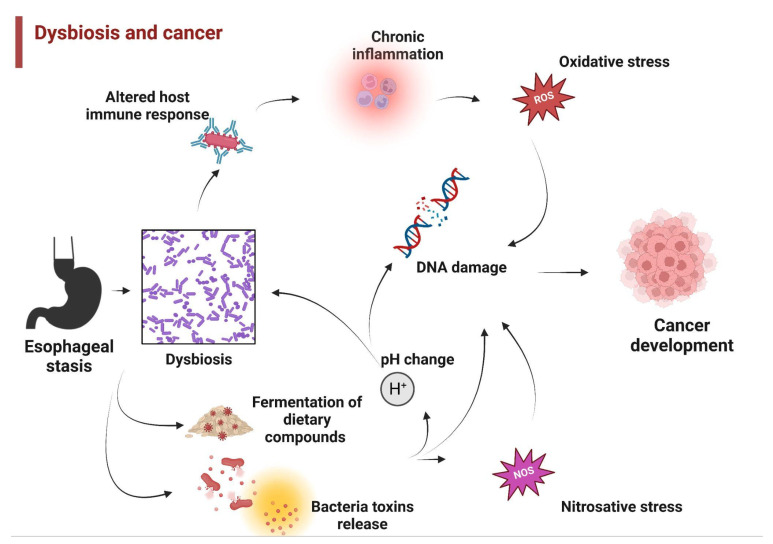
Dysbiosis in achalasia might be associated with cancer development through several mechanisms, including altered host immune response, chronic inflammation, carcinogenic metabolites released from bacteria and diet, and pH change.

Studies have shown that the gut microbiome plays a key role in metabolism, especially considering the risk factors that eventually may lead to the development of upper gastrointestinal tract cancer [10]. Dysbiosis may induce carcinogenesis through several mechanisms. See Figure 1.

Dysbiosis can lead to chronic inflammation and an altered immune response, both known to contribute to the development and progression of cancer [11]. Certain bacterial species associated with dysbiosis can trigger an inflammatory response, damaging cells, promoting DNA and chromosomal damage, and eventually promoting dysplasia and cancer development and progression [12].

Dysbiotic microbial communities can also produce metabolites with genotoxic properties that can damage DNA and promote carcinogenic mutations [13]. Also, some bacteria can produce enzymes that convert dietary compounds into carcinogenic substances or even generate reactive oxygen species that impose DNA damage [14].

The gastrointestinal endoluminal microbiota plays a crucial role in maintaining the integrity of the intestinal barrier, which can also be compromised by dysbiosis, allowing the overgrowth of certain strains of microorganisms and shrinkage of other groups, increasing toxins and pro-inflammatory molecules that translocate into the body [15]. Dysbiosis can trigger systemic inflammation and potentially promote the development of various cancers [10].

Microbes in the gut can influence host metabolism by participating in the breakdown and fermentation of dietary components. Dysbiosis can disrupt these metabolic processes, leading to the production of metabolites that can affect host cells and contribute to carcinogenesis. Additionally, dysbiosis can impair the immune system’s ability to detect and eliminate cancer cells, allowing tumors to evade immune surveillance [11].

A recently published systematic review suggests a potential correlation between esophageal microbiota and the response of tumors to neoadjuvant chemotherapy [16]. Specifically, high levels of intratumoral Fusobacterium nucleatum were associated with a poor response. No direct association between esophageal microbiota and the occurrence of complications was observed based on one study. High levels of intratumoral F. nucleatum, low abundance of Proteobacteria, and high quantity of Prevotella and Streptococcus species were correlated with shortened long-term survival in patients with esophageal cancer [16].

Moreover, food stasis can promote fermentation, release nitric oxide radicals, and induce chronic esophagitis, eventually leading to bacterial growth and epithelial dysplasia [17]. Bacteria present in the stasis liquid catalyze the production of nitrites, which are carcinogenic molecules [17]. In a prospective study, Pakecki et al. [18] analyzed the microbiota of patients with achalasia. The bacterial flora found in patients’ stasis fluid was constituted of microorganisms from the normal microbiota of the mouth and oropharynx, which proliferate in a medium rich in nutrients, neutral pH, and low oxidation-reduction potential, favoring the growth of anaerobic microorganisms. The bacteria overgrowth in those patients creates a favorable environment for the diet compounds reduction of nitrates and massive formation of nitrites and N-nitroso compounds. Additionally, chronic stasis makes these compounds remain for an extended period in the lumen. This theory explains the high incidence of cancer in more advanced megaesophagus.

Lipopolysaccharide (LPS) also might contribute to carcinogenesis in esophageal stasis. LPS is one of the main components of the gram-negative bacilli cell wall and a common proinflammatory factor that has been reportedly linked to the proliferation and migration of tumors [19,20]. LPS may regulate the induction and maintenance of stemness of tumor cells, promote metastasis, and induce therapeutic tolerance.

Xu et al. [19] investigated the significance of LPS stimulation in squamous cell carcinoma by collecting tissue samples from tumors and normal esophageal cell epithelium. The conclusion is that LPS acts as a tumor-promoting factor in squamous cell carcinoma by its cascade activation in a multi-step signaling axis related to the LPS-TET3-HOXB2 gene [19].

Peng et al. [21], in a case–control study, showed that high levels of LPS have a critical effect on the occurrence and development of esophageal cancer [21]. LPS treatment induces an increase in the levels of TLR4 and NF-κB. The authors found that the levels of LPS were higher in serum and fresh stool samples from esophageal cancer patients.

Functional experiments using the CCK8 assay and transwell assay showed that LPS contributed to the proliferation, migration, and invasion of EC109 cells, the human esophageal squamous cell carcinoma cell line [21]. Moreover, LPS induces EC109 to secrete IL-6 and TGF-beta 1, both known to be related to chronic inflammation and cell proliferation, respectively [22,23].

#### 3.1.2. Endoluminal pH and Carcinogenesis

The pH plays a crucial role in shaping the composition and function of microbial communities in different body sites [24]. Different microbial species thrive within specific pH ranges. The pH of a particular environment can influence the growth and survival of certain bacteria strains, while inhibiting others [25]. Any disruption in pH can lead to dysbiosis, where pathogenic or harmful bacteria may overgrow, compromising the microbial balance. Consequently, the change in pH in the achalasia esophageal lumen may be related to carcinogenesis.

The metabolites produced from bacteria fermentation also induce significant changes in pH. Dysbiosis can lead to a shift in pH, creating an environment that is more favorable for the growth of pathogenic microbes. A shift in pH leads to a change in bacteria, which in turn may change the pH, creating a vicious cycle until an equilibrium is reached [26].

The pH influences microbial metabolism and the production of metabolic byproducts. Many microorganisms produce acids or alkalis as byproducts of their processes, which can further affect the pH of the surrounding environment. Dysbiosis can lead to an imbalance in the production of these metabolites [27]. In addition, it may increase certain bacterial species that produce metabolites, like short-chain fatty acids (SCFAs). The pH within the gut influences the production of SCFAs, which play essential roles in gut health, immune modulation, and energy metabolism [28].

The pH of certain body sites is closely linked to maintaining mucosal integrity and immune response. Mucosal surfaces, like the gastrointestinal tract, have specific pH gradients essential for normal function and defense against pathogens. Dysbiosis-induced pH alterations can compromise the integrity of the mucosal barrier and affect the local immune response, increasing susceptibility to infections and chronic inflammation [15].

Additionally, pH change might have a significant role not only in cancer initiation, but also in cancer progression. The pH within the tumor microenvironment can profoundly impact cancer progression. Tumors often have an acidic microenvironment, primarily due to the increased lactic acid production as a byproduct of anaerobic metabolism in rapidly proliferating cancer cells. Acidic pH promotes tumor invasion, angiogenesis, and the ability of cancer cells to resist specific treatments, such as chemotherapy and radiation therapy [29].

Altered pH levels can influence cancer cell metabolism and their ability to adapt to changing conditions. Cancer cells can undergo metabolic reprogramming to thrive in acidic environments. They may switch to glycolysis (a less efficient form of energy production) to sustain their growth and survival [29]. This metabolic adaptation can confer a survival advantage on cancer cells and contribute to tumor progression.

The endoluminal pH can also impact the immune response against cancer cells. In an acidic tumor microenvironment, immune cells may become less effective in recognizing and eliminating cancer cells. Additionally, acidic pH can impair the function of immune cells, including T cells, natural killer cells, and antigen-presenting cells [30]. The weakened immune response can allow cancer cells to evade immune surveillance and promote tumor growth and metastasis.

Lastly, pH can influence genomic stability and the occurrence of DNA mutations. Acidic conditions can increase DNA damage and genetic instability, essential factors in cancer development. Altered pH can also affect DNA repair mechanisms, further contributing to genetic abnormalities and cancer progression accumulation [31].

There are few consistent study results in the literature regarding achalasia and subsequent pH disorders and their correlation with carcinogenesis. Crookes et al. [32] demonstrated in an in vitro study that chewed food with saliva at body temperature undergoes lactobacilli fermentation and generates lactic acid, leading to below 4.0 pH measures. Moreover, the lack of peristalsis and further poor acid clearance leads to prolonged contact between gastric reflux and esophageal mucosa and a pH drop.

The drop in pH levels in pH meter tests leads to the often common misdiagnosis of gastroesophageal reflux disease (GERD) in achalasia patients. In true GERD, the drop in pH level (pH 1.0 to 2.0) occurs abruptly, and it rapidly returns to physiological levels (pH around 6.5) [B], whereas in food fermentation, the drop in pH is slow (around six hours), usually at night, and usually does not reach levels below 3.0 [33].

Unfortunately, no current studies evaluate the role of pH change in achalasia and cancer. However, there are some studies evaluating the role of pH in carcinogenesis in GERD. In that way, we may hypothesize and extrapolate conclusions for achalasia.

Gastroesophageal reflux disease (GERD) is a chronic condition resulting from the retrograde flow of gastroduodenal contents into the esophagus and adjacent organs, causing a variable spectrum of symptoms (esophageal or extra-esophageal), with or without tissue damage. Decreased anti-reflux mechanisms and prolonged exposure to gastric juice can injure and irritate the esophagus, with acid being the primary determinant of esophagitis and reflux symptoms [24].

The epithelial layer of the esophageal mucosa is typically moist with saliva along its entire length, resulting in a pH that remains around 7 in normal individuals, providing a favorable condition for a variety of microorganisms. However, gastric material reflux can cause a sudden drop in pH values, reaching as low as 2. When chronic, it can result in mucosal damage and induce microenvironmental variations [34].

More recently, knowledge regarding microbiota composition in healthy individuals and individuals with esophageal diseases has been described in the literature through metagenomic-based investigations.

When analyzing the prevalence of bacterial agents in healthy esophageal mucosa, the presence of Streptococcus spp. has been consistently reported, often in lower proportions, along with Prevotella, Fusobacterium, and Veillonella [34]. In patients with conditions associated with chronic reflux of acidic gastric material into the esophagus, the esophageal microbiota is expected to be modified due to the decreased susceptibility of many species to acidic pH and bile salts. The presence of organisms, such as Veillonella, Prevotella, Haemophilus, Neisseria, and Campylobacter, is associated with esophageal mucosa in cases of reflux esophagitis related to GERD [34]. The microbiome profile changes if the patient is under acid reflux treatment [35].

### 3.2. Cancer Prevention

Considering the tight relationship between esophageal endoluminal dysbiosis and cancer development and progression, the next step is identifying potential cancer prevention measures targeting the microbiota.

#### 3.2.1. Could Antibiotics Work as Chemoprophylaxis for Esophageal Cancer?

Antibiotics are currently being studied as chemoprophylaxis not only in the development or progression of esophageal cancer, but also in the outcome of its treatment [16]. Chemoprophylaxis with antibiotics would involve targeting and potentially modulating the microbiota to reduce the risk or progression of esophageal cancer. As mentioned, in achalasia, there is a consequent change in the microbiota, as well as a change in the local pH, both favoring carcinogenesis. Therefore, it can be hypothesized that controlling certain bacterial strains with certain antibiotics will reduce carcinogenesis.

Small-spectrum antibiotics can eradicate or restrain undesirable microorganisms, while probiotics may introduce essential microbial elements. On the other hand, prebiotics serve as functional food ingredients that can modify the composition and behavior of the gastrointestinal microbiota by promoting the growth of beneficial microbes [16].

Nevertheless, the use of antibiotics for chemoprophylaxis in esophageal cancer is still debatable, as its effectiveness and safety profile are not yet well established. Some studies have indicated a potential link between specific bacterial species or dysbiotic states and esophageal cancer, suggesting that targeted antibiotic interventions would be beneficial. However, more research is needed to better understand the complex interactions among the microbiota, host factors, and cancer development before definitive recommendations.

It is important to consider that antibiotics not only target harmful bacteria, but also disrupt bacteria’s beneficial contribution. This disruption can lead to antibiotic resistance, altered microbial diversity, and potential side effects, requiring a cautionary approach. Several studies have demonstrated the negative impact of antibiotics on both the male and female reproductive systems, especially for macrolides [36]. Furthermore, based on the context of achalasia, it is known that oral drug absorption is erratic due to the prolonged esophageal transport time [37], and consequently, keeping a certain level of concentration of systemic or endoluminal antibiotics may be difficult.

In synthesis, while chemoprophylaxis with antibiotics for esophageal cancer is intriguing, its clinical application is still uncertain. Further research is needed to elucidate the role of esophageal dysbiosis in cancer development, identify specific microbial targets, and assess the long-term effects and safety of antibiotic interventions.

#### 3.2.2. Could COX Inhibitors Work as Chemoprevention for Esophageal Cancer?

Cox inhibitors, also known as cyclooxygenase inhibitors, are a class of medications that inhibit the activity of the cyclooxygenase enzymes COX-1 and COX-2 [38,39]. These enzymes are involved in the production of prostaglandins, which play a role in inflammation, pain, and other physiological processes. Additionally, COX inhibitors can inhibit angiogenesis (formation of new blood vessels) and modulate the immune response, contributing to their anticancer properties [38,39]. Previous studies have shown that Cox inhibitors can impact the composition of the gastrointestinal microbiota and change the global individual physiological status, influencing microbiota composition [40]. Consequently, Cox inhibitors could act through several mechanisms to prevent cancer development in high-risk groups. The role of COX inhibitors in cancer prevention has been extensively studied.

Using Cox inhibitors as chemoprophylaxis has shown significant benefits in reducing the risk of colorectal cancer [41]. The use of nonsteroidal anti-inflammatory drugs (NSAIDs), such as aspirin and celecoxib, has been associated with a decreased incidence of colorectal cancer, particularly in individuals at high risk, such as those with a history of adenomatous polyps or hereditary colorectal cancer syndromes. Certain studies have suggested that long-term use of low-dose aspirin may lower the risk of colorectal cancer, even in the general population [41].

In a randomized controlled trial, Baron et al. [42] evaluated the effect of aspirin and folate supplementation on the recurrence of colorectal adenomas, precursors of colorectal cancer. The authors demonstrated that aspirin (81 mg/day) reduced the recurrence of advanced adenomas by 19% after three years of treatment.

Bertagnolli et al. [43] investigated the selective COX-2 inhibitor celecoxib use in individuals with familial adenomatous polyposis, an inherited condition associated with a high risk of colorectal cancer. The study found a significant 22.5% reduction in the occurrence of advanced adenomas and colorectal cancer in individuals treated with celecoxib.

McNeil et al. [44] investigated the use of low-dose aspirin (100 mg/day) in individuals aged 70 years or older. While the primary outcome of major cardiovascular events did not significantly benefit, the secondary analysis revealed a 19% reduction in colorectal cancer incidence among those assigned to aspirin.

The risk reduction has been well demonstrated among patients with hereditary colorectal cancer. A long-term follow-up study among patients with Lynch syndrome found that 600 mg aspirin daily for at least two years significantly reduces the risk of future cancer [45].

Some studies also observed a significant role of the Cox inhibitors for esophageal cancer. Vaughan et al. [46] examined the association between some nonsteroidal anti-inflammatory drug use and the risk of neoplastic progression in Barrett’ s esophagus. The findings suggested that long-term use of nonsteroidal anti-inflammatory drugs might be associated with a reduced risk of esophageal adenocarcinoma.

Liu et al. [47], in an in vivo study, investigated the association between meloxicam use, a Cox-2 inhibitor, and the pathological findings in squamous cell carcinoma specimens. The authors found a significantly higher number of apoptotic cells in patients’ tumors using meloxicam.

Van Staalduinen et al. [48] showed a possible association between post-diagnosis aspirin use and overall survival in patients with esophageal cancer. The survival gain was consistent among different subgroups and both squamous and adenocarcinoma.

The aspirin antitumor mechanisms are not yet fully understood. Some accepted theories involve COX-mediated and COX-independent pathways, promoting the inhibition of chronic inflammation. A chronically inflamed system can activate specific transcription factors enrolled in esophageal carcinogenesis by affecting the activities of NF-κB and MAPK [39]. Furthermore, COX-2-derived prostaglandin E-2 (PGE-2) promotes the proliferation and apoptosis of cancer cells through different molecular pathways, including MAPK, PII3K, and cAMP/PK pathways. Therefore, aspirin may also interrupt cancer cell proliferation through these interrelated signaling pathways [38]. The presence of mutation of PIK3CA appears to favor aspirin’ s antitumor activity. This finding was first described in colorectal cancer [49] and is now established in esophageal cancer. Moreover, heparanase-based anti-metastatic and anti-angiogenic activities of aspirin may contribute to explaining its role as adjuvant treatment in patients already having cancer other than as a primary preventive method [38].

Finally, some studies have found that the Cox pathways are influenced by certain bacteria strains that release some prostaglandins and other mediators that act in the tumor microenvironment, influencing tumor immunity [50]. A study investigated batch incubations of human fecal microbiota. In this study, microbiota exposed to Cox inhibitors shifted their metabolic activity and composition [51]. Also, Cox inhibitors reduced butyrate production, suggesting that these drugs might reduce fermentation and its carcinogenic products.

Despite the potential benefits, Cox inhibitors can have side effects, including gastrointestinal complications, such as stomach ulcers, bleeding, and cardiovascular risks. Despite some studies that have found no major aspirin-related bleeding within an antitumor drug protocol [44,52], choosing Cox inhibitors for cancer prevention should be carefully weighed against the potential risks and individual factors, such as age, medical history, and concurrent medications. It is essential to consult with healthcare professionals to stratify an individual’ s overall risk–benefit profile and determine the most appropriate approach. The aspirin dosage for antitumor use, an essential point for both the antitumor effects and risk of bleeding, remains controversial and differs among studies [38]. Further research should be conducted to determine a proper dose.

In patients with achalasia, the bleeding risk is even higher due to impaired esophageal emptying, which may lead to erratic oral drug absorption, promoting periods of underdose and overdose. Considering the potential benefit and risks of using Cox inhibitors in preventing esophageal cancer, these drugs should only be considered in research protocols for chemoprophylaxis for cancer in achalasia. Succedaneum is a safer aspirin (NOSH-aspirin) with fewer gastrointestinal side effects and less risk for bleeding when compared to aspirin [38]. Future studies might investigate the role of NOSH-aspirin as chemoprophylaxis for esophageal cancer risk groups.

#### 3.2.3. Does Myotomy Reduce the Risk for Esophageal Cancer?

In patients with achalasia, chronic food accumulation leads to bacteria dysbiosis. Some recent studies showed that in these patients’ esophagus, a lactic acid-producing bacterium (*Lactobacillus)* is present in greater amounts. The more severe the disease is, the higher the abundance of these bacteria [53].

In this scenario, cancer development is believed to be related to the production of pro-inflammatory substances, carcinogenic metabolites, and the alteration of the host immune surveillance function by gastrointestinal microbiota. Lactic acid-producing bacteria can perform nitrate reduction, leading to an acid environment. These changes could lead to mutagenesis and angiogenesis and inhibit apoptosis [54].

The mechanisms of carcinogenesis are not entirely understood. Still, if we consider that food stasis promotes the proliferation of bacteria, leading to cellular changes causing mutagenesis, angiogenesis, and inhibition of apoptosis, the intervention in achalasia may relieve chronic retention and decrease the risk of carcinogenesis [55].

In a systematic review and meta-analysis, Tustumi et al. [56] evaluated the risk for cancer development in achalasia after cardia myotomy. The authors found that the risk for cancer development increases over time, even after myotomy. Consequently, currently, there is no evidence that myotomy would reduce the risk for cancer development, despite the theoretical amelioration of food stasis and fermentation. However, this systematic review did not conclude that cardia myotomy could not reduce the risk for cancer development since no controlled study is available evaluating the role of myotomy in cancer prevention. Additionally, this meta-analysis did not include peroral endoscopic myotomy.

Peroral endoscopic myotomy (POEM) is an evolving technique to treat achalasia. It consists of four steps: esophageal mucosal incision and access to the submucosal space; creation of a submucosal tunnel; incision of the esophageal muscles (myotomy); and closure of the mucosal incision. POEM is a minimally invasive procedure that is performed under direct endoscopic vision without the need for hospitalization. The success rate up to 12 months is 82.4% [57]. POEM and laparoscopic Heller’s myotomy have similar efficacy for post-operative symptoms control and adverse events. However, POEM has shown favorable long-term efficacy with less invasiveness [58].

Few studies evaluate esophageal microbiota before and after myotomy in achalasia. Takahashi et al. [58] performed a metagenome sequencing to identify the main bacteria strains before and after POEM in achalasia. The authors used endoscopic brushing to collect oral and esophageal mucosa swabs. The available data did not show a significant difference in the oral microbiota before or after POEM. However, there was a substantial increase in esophageal *Haemophilus* and *Neisseria* after POEM, and the overall inflammation and the number of esophageal epithelial nuclei and Ki-67-positive cells decreased after POEM. Hence, it might reduce the risk of esophageal carcinogenesis by improving food stasis and inflammation. Takahashi et al. evaluated the microbiome after two months of POEM. Perhaps microbiome changes may be enhanced in the long term [59].

However, another study [53] could not show significant alterations of the esophageal microbiota in patients with achalasia pre- and eight weeks post-POEM. Although POEM treatment improves esophageal clearance, the esophageal microbiota in patients with achalasia can stay the same due to remaining food stasis, even to a small extent [53].

There are few studies on this topic, and most of them did not investigate microbiota change before and after myotomy and disease-specific characteristics of the oral and esophageal microbiota in patients with achalasia and cancer. Therefore, further research is needed to demonstrate whether the esophageal microbiota in patients with achalasia shifts after myotomy and if it can prevent esophageal cancer development [58].

## 4. Discussion

Given the data presented in this critical review, it can be concluded that there is a lack of studies, mainly clinical trials, in the current medical literature to understand better the relationship between the esophageal microbiome and carcinogenesis, as well as its influencing factors discussed in this article, especially in achalasia.

Only some research has been conducted on esophagus microbiota and cardia myotomy. Hardly any studies compare POEM and laparoscopic Heller’s myotomy for dysbiosis control. Most of the published studies in the scientific community have a significant risk of bias and are far too heterogeneous, lacking long-term follow-up results [55]. It is necessary to access larger-scale multicenter studies where POEM is compared with other standard procedures, including surgical myotomy, in a randomized manner, comparing the efficacy of these interventions in cancer prevention.

Future studies of bacterial and neoplasm biomarkers may point to precision therapies and chemoprophylaxis strategies. Considering that achalasia can be considered a high-risk condition for cancer, further investigations are needed to elucidate dysbiosis’ s carcinogenic mechanisms and identify potential therapeutic or preventive interventions targeting the microbiota.

## Data Availability

Data are available upon reasonable request to the corresponding author.
